# The Role of Glucagon-Like Peptide-1 (GLP-1) Receptor Agonists in Acute Cholecystitis After a Routine Colonoscopy: A Case Report

**DOI:** 10.7759/cureus.79105

**Published:** 2025-02-16

**Authors:** Ahmad Abdulraheem, Dania Shukri, Nadera Altork, Usman Afzal, Mohammed Abu-Rumaileh, Alireza Meighani

**Affiliations:** 1 Internal Medicine, MedStar Washington Hospital Center, Washington, D.C., USA; 2 Internal Medicine, Jordan University Hospital, Amman, JOR; 3 Internal Medicine, The University of Toledo, Toledo, USA; 4 Gastroenterology and Hepatology, MedStar Washington Hospital Center, Washington, D.C., USA

**Keywords:** acute cholecystitis, colonoscopy complications, glp-1 receptor agonist, pericholecystic fluid, semaglutide

## Abstract

Colonoscopy (CLN) is a common procedure for colon cancer screening and diagnosing various conditions. Acute cholecystitis (AC), though rare, has been reported as a complication. We present a 66-year-old female on semaglutide for obesity who developed AC within 72 hours post-CLN. Considering the increasing use of glucagon-like peptide-1 receptor agonists (GLP-1 RAs) and their impact on gallbladder motility, these medications may contribute to this complication. Future research is crucial to investigate whether a washout period for GLP-1 agonists before CLN is needed to reduce the risk of AC, if such a risk exists.

## Introduction

Colonoscopy (CLN) is a common procedure performed to investigate or treat various gastrointestinal (GI) conditions. Around 2.5-11% of patients may experience mild discomfort afterward, often due to endoscopic looping and the application of manual pressure [1]. Severe complications are rare, with bowel perforation being the most common, occurring in less than 0.3% of cases [2]. Other rare complications include bleeding, splenic rupture, acute appendicitis, diverticulitis, and acute cholecystitis (AC) [2,3]. Only a few cases worldwide have been reported AC following a CLN.

Notably, Milman and Goldenberg reported the first two instances of AC following a CLN [4]. In most reported cases, these complications manifested within 72 hours, with imaging studies revealing the presence of gallstones [2]. 

Glucagon-like peptide-1 receptor agonists (GLP-1 RAs) have been widely used for glycemic control in patients with type 2 diabetes, cardiovascular mortality benefit, and weight loss. However, they have adverse effects on the GI tract motility. Woronow et al. and He et al. studied the increased risk of gallbladder and biliary system disease in patients on GLP-1 RA, especially with greater duration and higher doses [5,6].

Recently, the American Society of Anesthesiologists published consensus-based guidance, due to the absence of strong clinical data, on the pre-operative management of patients treated with GLP-1 RAs. The guidance recommends holding GLP-1 for one day in patients on daily-dosed medications and for one week in those on weekly-dosed medications prior to an elective procedure, given the potentially high risk of aspiration and anesthesia-related complications [7]. On the other hand, the American Gastroenterological Association (AGA) recommended an individualized approach for managing patients on GLP-1 RAs before endoscopy considering beforehand the reason for GLP-1 RA use, as discontinuation may pose more risk than benefit [8].

This case highlights a case report having AC after a routine CLN while taking semaglutide for obesity, and she stopped it one week before the CLN. 

## Case presentation

A 66-year-old female with a history of hypertension on amlodipine 5 mg daily and obesity, on the maximum dose of semaglutide (2.4 mg subcutaneous injection weekly), presented to the hospital complaining of periumbilical, sharp, non-radiating pain associated with vomiting that started one day after undergoing a screening CLN at an outside hospital. She consumed a small amount of food post-CLN, after which she began experiencing symptoms. Based on the procedure note, the CLN was uneventful and revealed only a small white patch in the rectum, and biopsy results showed normal tissue. The patient had a history of an uneventful screening CLN more than five years ago, and had been advised to withhold her semaglutide dose one week before the CLN due to its potential to cause delayed gastric emptying. She was not on any other medications, including opioids.

The patient initially presented to an urgent clinic, where an abdominal X-ray was unremarkable. As her pain continued to worsen, she sought evaluation in the emergency department. She had elevated blood pressure, tachycardia, and tenderness in the right upper quadrant on examination. Labs revealed leukocytosis (17.7k) with a neutrophilic shift, aspartate aminotransferase (AST) 26 IU/L, alanine aminotransferase (ALT) 18 IU/L, total bilirubin 1.0 mg/dl and alkaline phosphatase 57 IU/L.

**Figure 1:**
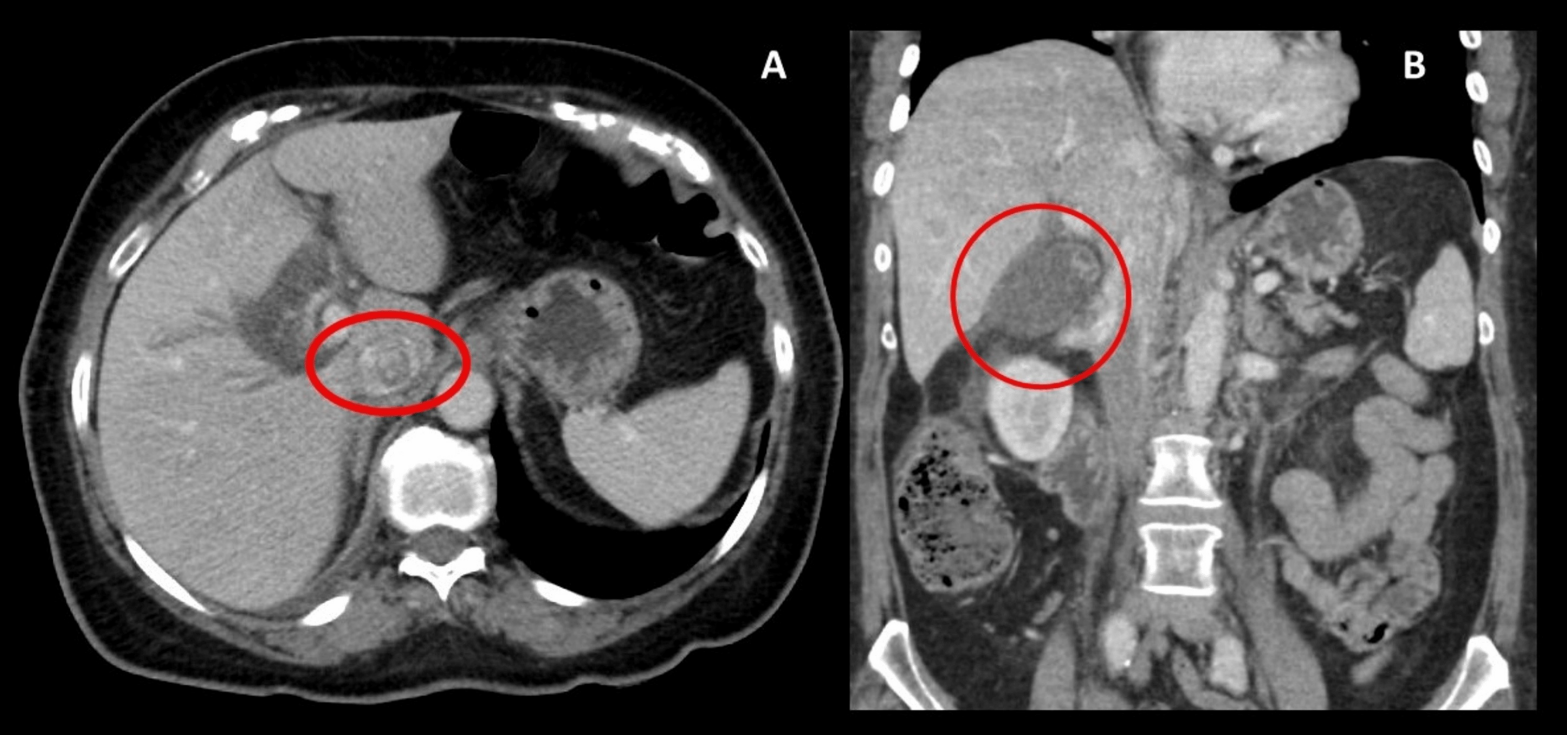
CT Abdomen showing distended GB, GS in the GB and extensive pericholecystic fat stranding. A- Cross sectional view. B- Coronal sectional view. CT: Computed tomography. GB: Gallbladder. GS: Gallstones

Liver function tests and urinalysis were normal. CT abdomen showed mild intrahepatic biliary ductal dilatation, a distended gallbladder with gallstones, and pericholecystic fat stranding, consistent with AC (Figure 1).

**Figure 2:**
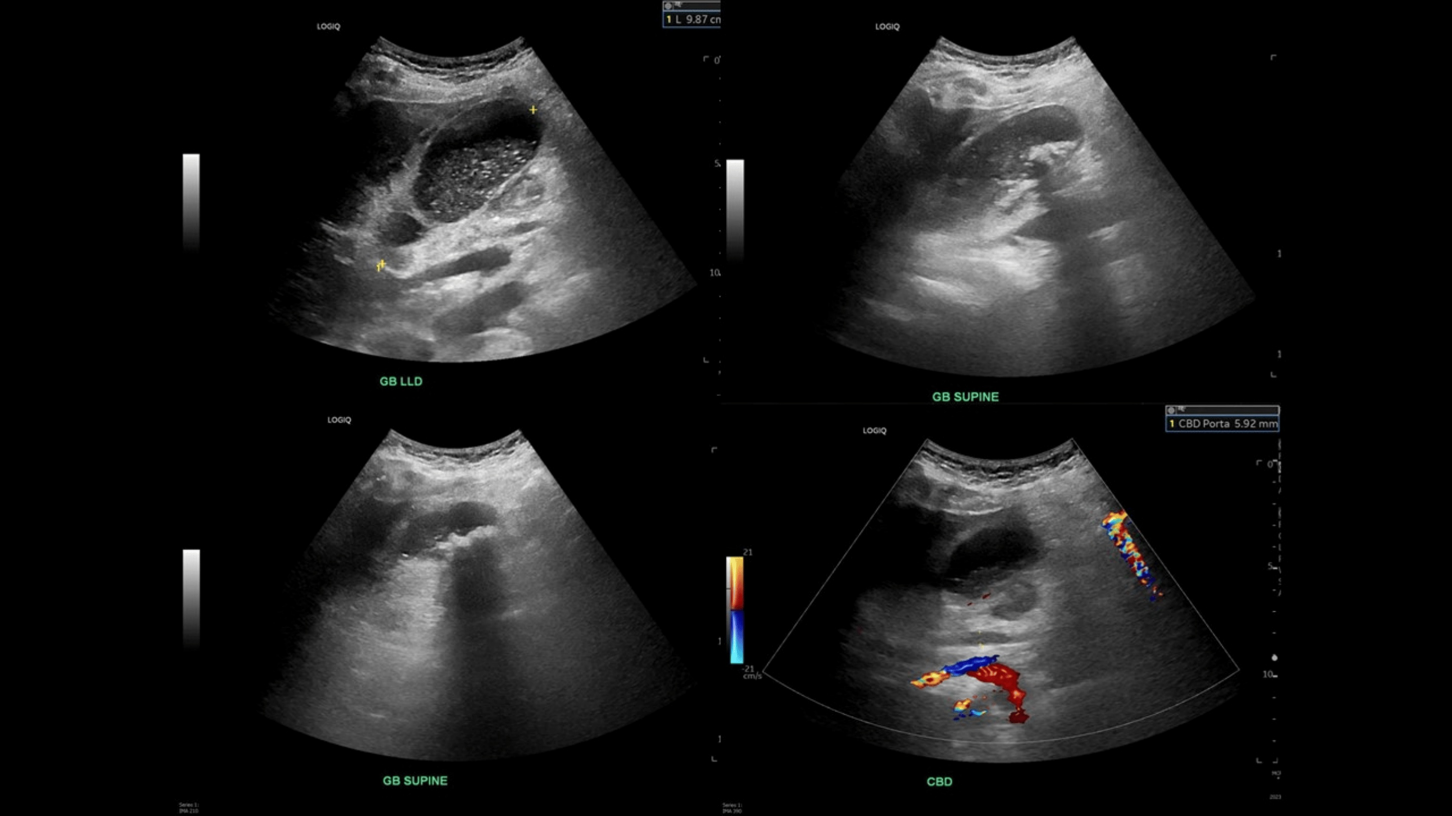
Abdominal ultrasound showing gallbladder stones and thickened gallbladder walls.

An abdominal ultrasound showed no biliary ductal dilatation, common bile duct (CBD) diameter 0.6 cm, however, the gallbladder was moderately distended with stones and sludge, and diffuse gallbladder wall edema with trace pericholecystic fluid and positive sonographic Murphy sign (Figure 2). General surgery was planned for laparoscopic cholecystectomy, but given CT findings, an interventional radiologist was consulted for cholecystostomy tube placement.

The patient showed clinical improvement in abdominal pain and leukocytosis with conservative treatment, including IV fluids and IV antibiotics. Consequently, cholecystostomy tube placement was deferred. The patient was discharged on semaglutide and later underwent laparoscopic cholecystectomy.

## Discussion

AC is commonly linked to biliary sludge or stones obstructing the CBD, particularly in patients with risk factors like obesity, pregnancy, or hemolytic anemia, leading to bile stasis and infection. However, its occurrence post-CLN remains unclear. Proposed mechanisms include dehydration from bowel preparation causing biliary stasis and gallbladder distension, especially in asymptomatic gallstone patients [3,9], bacterial translocation due to abdominal pressure during challenging procedures [9,10], and gallstone displacement into the biliary system by scope movement [9].

Fernandez-Martinez et al. and Campbell et al. reported extracolonic bacterial proliferation, including Clostridium spp., Enterococcus faecalis, and beta-lactamase-producing Escherichia coli, in pericholecystic fluid post-CLN, supporting bacterial translocation [10,11]. Warfe et al. highlighted excessive organ manipulation during CLN as a factor in cholecystitis, describing gallbladder twisting due to navigating a tortuous colon [12]. Additionally, case reports suggest that dehydration from bowel preparation may increase bile lithogenicity, potentially triggering AC.

A systematic review and meta-analysis by Liyun et al. found an increased risk of gallbladder and biliary diseases with GLP-1 RAs, particularly at higher doses, longer durations, and for weight loss [5,6]. Since higher doses are often prescribed for weight loss rather than type 2 diabetes as in our case, the risk may vary with dosage [5].

The World Health Organization-Uppsala Monitoring Centre (WHO-UMC) causality assessment classifies the reaction as "Possible," based on its temporal association (occurrence within a 72-hour timeframe) with the drug [13]. However, other contributing factors, such as dehydration, fasting, and procedural stress, along with the known effects of GLP-1 receptor agonists on gallbladder motility, could also explain the event. Similarly, the Naranjo Scale assessment resulted in a "Possible" classification (Score: 2-4), as the reaction occurred after drug administration, but alternative explanations exist [13]. Rechallenge and dechallenge data were unavailable to further assess causality, as the gallbladder was surgically removed. This rationale underscores the likelihood of this unusual adverse event pattern, suggesting that it might be more than mere coincidence.

AC following CLN is a notable concern in patients with abdominal pain and vomiting, particularly among GLP-1 RA users, given the medications' association with gastrointestinal side effects. With the increasing use of GLP-1 RAs, the potential for post-CLN cholecystitis warrants attention. An abdominal X-ray is recommended to rule out bowel perforation, and a low threshold for abdominal ultrasound is advised to assess for possible AC in the appropriate clinical context.

## Conclusions

In the context of the increasing use of GLP-1 agonists for various indications, it is crucial to monitor potential side effects to prevent life-threatening complications, particularly during pre-operative evaluations. Although our patient stopped taking semaglutide (a long-acting GLP-1 RA) one week before the procedure, she unfortunately developed AC. This suggests that while AC after a CLN is typically rare, the risk may be elevated in the presence of recent high-dose GLP-1 RA use. Further research and documentation of similar cases are necessary to assess the need for a washout period for GLP-1 RAs before CLN. In the meantime, ensuring adequate patient hydration to reduce biliary stasis is essential. Additionally, risk stratification should be considered for patients with a history of gallbladder stones.
